# Novel Antihypertensive Peptides Derived from Adlay (*Coix larchryma-jobi* L. var. *ma-yuen Stapf*) Glutelin

**DOI:** 10.3390/molecules22010123

**Published:** 2017-01-13

**Authors:** Bin Li, Liansheng Qiao, Lingling Li, Yanling Zhang, Kai Li, Lingzhi Wang, Yanjiang Qiao

**Affiliations:** Beijing University of Chinese Medicine, 6 South Zhonghuan Road, Wangjing, Chaoyang District, Beijing 100102, China; libinzyy@163.com (B.L.); b20100222012@163.com (L.Q.); lilingling@126.com (L.L.); collean_zhang@163.com (Y.Z.); lk20162016@163.com (K.L.)

**Keywords:** *Coix larchryma-jobi* L. var. *ma-yuen Stapf*, glutelin, angiotensin I-converting enzyme, antihypertensive peptide, molecular simulation

## Abstract

Our previous studies have shown that *Coix* glutelin pepsin hydrolysate can effectively inhibit angiotensin converting enzyme (ACE) activity in vitro. The main purpose of this study was to obtain potent anti-hypertensive peptides from *Coix* glutelin. The *Coix* glutelin hydrolysates (CGH) were prepared by pepsin catalysis and further separated by an ultrafitration (UF) system, gel filtration chromatography (GFC) and reversed-phase high performance liquid chromatography (RP-HPLC). As a result, the sub-fraction F5-3 had the highest ACE-inhibitory activity. Six ACE inhibitory peptides were identified using nano-liquid chromatography coupled to tandem mass spectrometry. The most potent peptide GAAGGAF (IC_50_ = 14.19 μmol·L^−1^) was finally obtained by further molecular simulation screening and a series of division and optimization. Single oral administration of synthesized GAAGGAF at 15 mg/kg body weight (BW) in spontaneously hypertensive rats (SHR) could reduce the systolic blood pressure (SBP) around 27.50 mmHg and the effect lasted for at least 8 h. The study demonstrated for the first time that the ACE inhibitory peptide GAAGGAF from *Coix* glutelin has a significant antihypertensive effect, and it could be a good natural ingredient for pharmaceuticals against hypertension and the related diseases.

## 1. Introduction

Adlay seed (soft-shelled Job’s tears, *Coix larchryma-jobi* L. var. *ma-yuen Stapf*) has been used in traditional Chinese medicine and has been a nourishing food for more than a thousand years. It has been used to treat warts, chapped skin, rheumatism, neuralgia, hypertension as well as being an anti-inflammatory and anticancer agent [1]. An increasing effort had been concentrated on finding active components from adlay. The coixenolide (I) isolated from the seeds of *Coix* was reported as having growth-inhibiting activity against Ehrlich ascites sarcoma cells in mice [2]. The main ingredient of Kanglaite injection (KLT), which has been widely used for cancer treatment, is *Coix* seed extract [3]. The ethyl acetate fraction from ethanolic extracts of adlaytesta had an inhibitory effect on allergic response [4]. Six compounds with high free radical scavenging activity were identified from adlay hull after the process of activity-directed fractionation and purification [5]. Protein is one of the important structural and functional units of life activities. *Coix* seeds are rich in protein and amino acids and have dual functions of being medicine and food. However, only a few studies have reported the functions of peptides from *Coix* [6]. Therefore, it is necessary to further characterize the peptides from *Coix* seeds.

Hypertension is highly prevalent and is a major risk factor for cardiovascular and renal diseases [7]. Angiotensin converting enzyme (ACE) is a key regulator in the renin-angiotension-aldosterone system (RAS) that converts an inactive form of angiotensin I to vasoconstrictor angiotensin II and also inactivates the catalytic function of bradykinin in the kallikrein-kimin system. Thus, inhibitors of ACE are widely used in the therapy for hypertension—for instance, captopril, enalapril and lisinopril. However, these synthetic drugs often cause some serious side effects including cough, loss of taste, renal impairment, and angioneurotic oedema [8]. Peptides with potent ACE inhibitory activity derived from natural food have great potential as antihypertensive agents [9].

A number of ACE inhibitory peptides have been isolated from a variety of traditional Chinese medicine protein hydrolysates. Our previous studies have shown that *Coix* glutelin hydrolysate (CGH) can effectively inhibit ACE activity in vitro [10]. The purpose of the current study was to fractionate the CGH and identify the peptides responsible for the potent ACE inhibitory activity. The identified peptides were then screened for their ACE inhibitory activities by molecular simulation, and further structural optimization by molecular docking. Meanwhile, the antihypertensive effect on spontaneously hypertensive rats (SHR) was also investigated.

## 2. Results

### 2.1. The Effect of the Ultrafiltration Pressure on the Glutelin Hydrolysate’s ACE Inhibitory Activity

The CGH with molecular weight less than 3 kDa was obtained by ultrafiltration (UF) under four different pressures. The corresponding ultrafiltrate was lyophilized, and its ACE inhibitory activity evaluated by the reversed phase high performance liquid chromatography (RP-HPLC) method. The ACE inhibitory rates for the ultrafiltrates obtained under four different pressures (0.1, 0.2, 0.3 and 0.4 Mpa) at the concentration of 0.02 mg·mL^−1^ were 44.76%, 36.69%, 39.35% and 37.70%, respectively. The hydrolysate obtained under 0.1 Mpa UF pressure exhibited the significantly highest (*p* < 0.05) inhibitory potential with the half maximal inhibitory concentration (IC_50_) of 46.70 ± 1.34 μg·mL^−1^. Therefore, the UF pressure of 0.1 Mpa was employed for the subsequent experiment.

### 2.2. GFC Isolation of ACE Inhibitory Peptides

**Figure 1:**
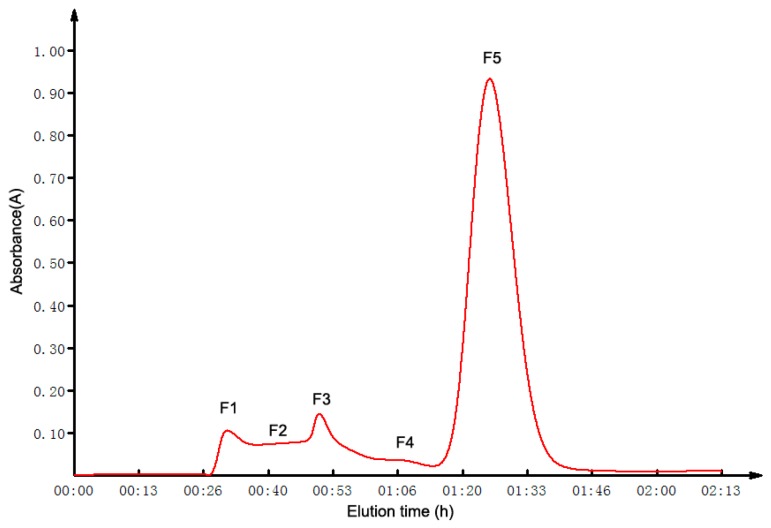
Gel filtration chromatography of the ultrafiltrate obtained under 0.1 Mpa pressure.

The ultrafiltrate obtained under 0.1 Mpa UF pressure was loaded into a Sephadex G-10 column for further separation. The chromatogram was presented in Figure 1. In total, five fractions (F1–F5) were collected, lyophilized and their ACE inhibitory activity evaluated for the final concentration of 0.02 mg·mL^−1^ (Supplementary Materials). All five of the fractions displayed ACE inhibitory activity to some extent. The fraction F5 exhibited the highest inhibitory activity (84.75% ± 0.42%), and the IC_50_ of it was 14.6 ± 0.58 μg·mL^−1^.

### 2.3. Purification of ACE Inhibitory Peptides by RP-HPLC

**Figure 2:**
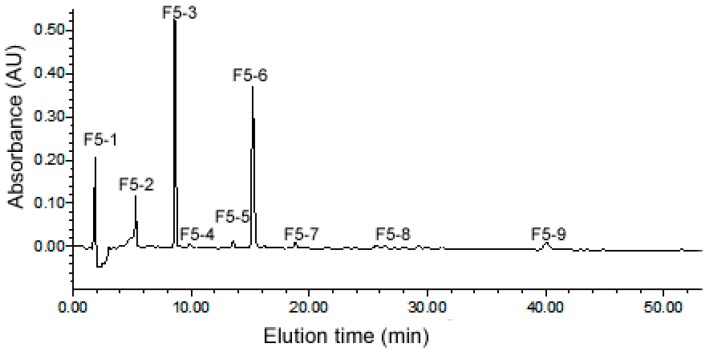
Purification profile of the fraction F5 by the reversed phase high performance liquid chromatography (RP-HPLC).

The fraction of F5 from gel filtration chromatography (GFC) was further purified by RP-HPLC on a SymmetryPrep C_18_ column using a linear gradient of 5%–30% acetonitrile (Figure 2). According to the figure, most of the peptides were eluted out within 40 min. A total of nine major sub-fractions (F5-1 to F5-9) were collected and the evaluation of their ACE inhibitory activity was conducted at the final concentration of 0.01 mg mL^−1^.There was significantly different (*p* < 0.05) ACE inhibitory activity among these sub-fractions. The sub-fraction F5-3 possessed the highest inhibitory rate (60.06%), while the lowest for F5-9 (9.16%).

### 2.4. Identification of Peptides from Sub-Fraction F5-3

The most potent sub-fraction F5-3 was analyzed by electrospray ionisation tandem mass spectrometry (ESI-MS/MS) based on a Triple TOF^TM^ 5600 system. In total, six peptide sequences were identified and they varied between 6 and 18 amino acids in length. The amino acid sequences, experimental and calculated Mr are listed in Table 1.

### 2.5. Molecular Simulation Screening and Further Optimization

Molecular simulation was then employed to estimate the ACE inhibitory potential of six identified peptides. The pharmacophore model of ACE inhibitors was first utilized to analyze the key pharmacodynamics and structural features of the peptides. The peptides matched with pharmacophore completely were further refined by molecular docking, and the binding mode and affinity between ACE and peptides were analyzed by docking. The -CDOCKER ENERGY of the initial ligand lisinopril was 71.22, which was regarded as the threshold value for evaluating the ACE inhibitory activity of peptides.

The pharmacophore mapping results showed that VGQLGGAAGGAF and QSGDQQEF had high ACE inhibitory potency. Then, the molecular docking simulation indicated that only VGQLGGAAGGAF was successfully docked, while part of the amino acids of it remained outside the active pocket of ACE. According to the characteristics, further optimization of VGQLGGAAGGAF was implemented by in silico proteolysis, by which four peptides were obtained (Table 2). Among the peptides, GGAAGGAF might have high ACE inhibitory activity. The pharmacophore mapping results showed that four pharmacophore features were all matched with the C-terminal of GGAAGGAF playing the main role, so the further sequential division from the N-terminus was carried out and six peptide fragments (No. 3-1~3-6) were obtained. All of the peptide fragments were analyzed by pharmacophore and molecular modeling studies. Finally, heptapeptide GAAGGAF with higher ACE inhibitory activity was obtained. The predicted results were showed in Table 2.

**Figure 3:**
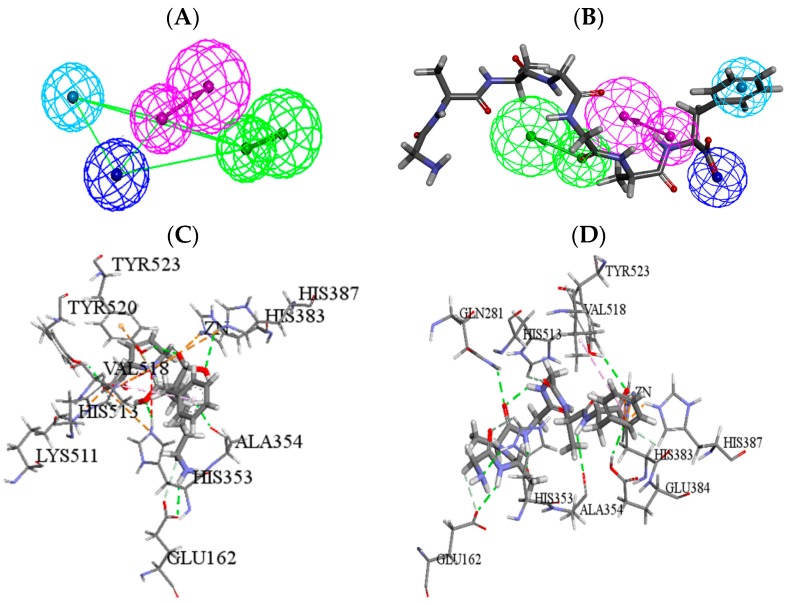
Representation of the angiotensin converting enzyme (ACE) pharmacophore model and molecular interactions between PDB 1O86 and the ligands. Purple sphere indicated hydrogen bond donor, green sphere indicated hydrogen bond acceptor, light blue sphere indicated hydrophobic aromatic features, and dark blue sphere indicated negative ionizable. (**A**) The best pharmacophore model of ACE; (**B**) The ACE pharmacophore model mapped with GAAGGAF; Molecular interactions between PDB 1O86 and the ligands: (**C**) Molecular interaction between 1O86 and lisinopril; and (**D**) Molecular interaction between 1O86 and GAAGGAF.

The mapping result of ACE pharmacophore model and GAAGGAF was showed in Figure 3. Then, the potential anti-hypertensive peptide GAAGGAF was docked into 1O86 using CDOCKER. Compared to the positive drug lisinopril, GAAGGAF had the higher score by pharmacophore and docking (Table 2). In addition, there were nine key residues which were consistent between lisinopril and GAAGGAF, including ZN701,GLU162, HIS353, ALA354, HIS383, HIS387, HIS513, VAL518, and TYR523 (Figure 3). The interaction types contained hydrogen bonds, electrostatic and hydrophobic action. GAAGGAF bound to the substrate-binding site of ACE with binding modes very similar to the initial compound lisinopril. Then, GAAGGAF was further synthesized and the IC_50_ value was obtained with 14.19 ± 0.89 μmol·L^−1^, according to the method described by Yuan et al. [10].

### 2.6. The Antihypertensive Effect of GAAGGAF In Vivo

**Figure 4:**
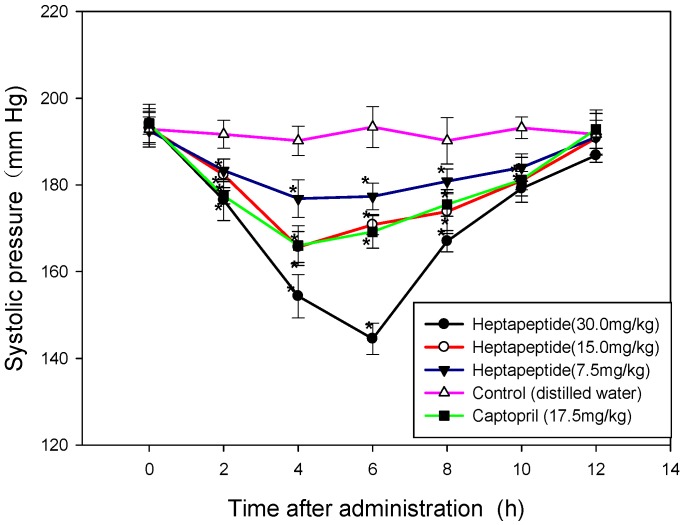
Change of systolic blood pressure (SBP) after oral administration of GAAGGAF in spontaneously hypertensive rats (SHR). Each point on the figure represented mean SBP of six animals and the vertical bars indicated the standard errors. Significant different from control group: * *p* < 0.05.

The antihypertensive effect of GAAGGAF was evaluated by measuring the change of systolic blood pressure (SBP) after oral administration. As shown in Figure 4, oral administration of water did not affect SBP in the negative control group, whereas administration of GAAGGAF decreased SBP in a dose-dependent manner. The SBP significantly decreased at 2, 4, 6, 8, 10 h after administration of this heptapeptide at the dose of 30.0 mg/kg BW. Maximum SBP reduction of 49.67 mmHg was observed 6 h after administration. For the middle dose (15.0 mg/kg BW) group, the SBP significantly decreased to 165.67 mmHg (reduction of 27.50 mmHg) at 4h after administration. The SBP in the low dosage GAAGGAF group was obviously reduced, although the antihypertensive effect was inferior to the higher dose groups. For the captopril group, the maximal decrease of approximately 28.17 mmHg was detected4 h after administration. The antihypertensive effect in SHRs administrated that GAAGGAF could persist for at least 8 h. In all cases, the SBP values were restored to their initial levels 12 h post administration.

## 3. Discussion

Natural source-derived ACE-inhibitory peptides have attracted widespread attention because of their fewer side effects. Since the first natural ACE inhibitory peptides were isolated from snake venom, a number of peptides inhibiting the activity of ACE have been obtained from the protein enzymatic hydrolysate from a variety of plants and animals. Ghassem et al. [11] has identified ACE inhibitory peptides VPAAPPK (IC_50_ = 0.45 μmol·L^−1^) and NGTWFEPP (IC_50_ = 0.63 μmol·L^−1^) from the hydrolysate of haruan myofibrillar protein by proteinase K and thermolysin. IIe-Val-Tyr was identified as a potential ACE inhibitor from the wheat germ hydrolyzate [12]. Recently, there have been three peptides (LY, TF and RALP) identified from rapeseed protein hydrolysate, which display moderate ACE inhibitory activity with IC_50_ values of 0.11 mmol·L^−1^, 0.81 mmol·L^−1^, 0.65 mmol·L^−1^, respectively [13]. From corn gluten meal, a new inhibitory peptide sequence (Ala-Tyr) was determined with the IC_50_ of 14.2 μM [14]. In this research, heptapeptide GAAGGAF derived from CGH was obtained and manifested similar in vitro inhibitory activity (IC_50_ = 14.19 μM).

A number of studies have reported that low molecular weight peptides are mainly associated with the ACE inhibitory activity [15]. Escudero et al. [16] reported that the most potent peptide AAATP was identified from Spanish dry-cured ham decreased SBP around 25.62 mmHg in SHRs after 8 h administration. Singal oral administration of AWLHPGAPKVF isolated from *P. esculenta* protein hydrolysate could reduce the SBP around 30 mmHg at 10 mg/kg dose with 8 h antihypertensive duration [17]. In this study, CGH (≤3 kDa) had the gratifying ACE-inhibitory activity with an IC_50_ value of 46.70 μg·mL^−1^, and six peptides were finally identified.

The quantitative structure-activity relationship research for ACE inhibitory peptides showed that hydrophobic amino acids, especially those with aliphatic chains such as Gly, Ile, Leu and Val are characteristic for the N-end of the peptide, while C-end are often identified to contain Try, Phe, Trp and pro [18,19]. Thus, our heptapeptide sequence with Gly N-end and Phe C-end is consistant with the active inhibitor feature structure. Molecular simulation is a time-saving way to estimate the ACE inhibitory ability of peptides and their bioactivity mechanisms [20]. In a molecular simulation modeling study, pharmacophore and molecular docking are commonly used methods to predict and evaluate the biological potency of the compounds [21]. Wang et al. [22] reported that ligand-based pharmacophore is a powerful method for screening ACE inhibitory peptides and three novel tripeptides (RSP, PPL and PGP) were obtained and biologically evaluated in vitro. Meanwhile, molecular docking has also been applied to identify ACE-inhibitory peptides with Pro C-terminus by Hai-Bang and Shimizu [23], which also indicated that aromatic residues in C-terminus are vital for the ACE inhibitors. The combination screening of pharmacophore and docking was implemented in our research, which could improve the screening efficiency and accuracy.

However, the positive correlation between the IC_50_ value of ACE in vitro and the antihypertensive effects of peptides in vivo is not always detected. For example, IY and RIY, two antihypertensive peptides isolated from rapeseed digestions, caused a reduction of systolic blood pressure in SHR of approximately 10 mmHg at a dosage of 7.5 mg/kg, although IY showed seven times more potent activity to inhibit ACE than the latter [24]. This means that alternative mechanisms of biopeptides are involved in in vivo antihypertensive activity. DNA microarray analysis of the gene expression in the aorta of SHR fed on VPP and IPP for five days indicated that the NO or prostanoids inducing vasodilation may be involved in cardiovascular function of these two tripeptides [25]. Ang-(1–7) induced vasodilatation and bradykinin-induced relaxations in rat mesenteric arteries were also augmented by IPP and Pro [26]. The milk casein hydrolyste product, which was administered orally in SHR at a dose of 800 mg/kg body weight for six weeks, could improve aorta and mesenteric acetylcholine relaxation and increase the eNOS expression in aortas of SHR. Meanwhile, a significant decrease was observed in interstitial fibrosis and left ventricular hypertrophy. However, the plasma ACE activity was surprisingly increased for the treated rats. Therefore, some other mechanisms beyond ACE inhibition are involved in the biopeptides [27]. Matsui et al. [28] reported that the di-peptide VY inhibited the proliferation of human vascular smooth muscle cells by the stimulation of a voltage-gated l-type Ca^2+^ channel agonist. Our results showed that GAAGGAF has the ACE inhibitory activity with IC_50_ value of 14.19 μmol·L^−1^ and the single oral administration (15 mg/kg BW) of it showed a significant antihypertensive activity which was similar to that of captopril. Therefore, in future research, cell models (human vascular endothelial cell and human vascular smooth muscle cell) and animal models will be applied to inspect the expression pattern of the RAS and other signal transduction pathways in transcription, translation and metabolism levels. The specific genes responsible for the *Coix* peptides may be detected and verified by overexpression as well as RNAi technology. Meanwhile, for coeliac disease patients, dietary gultelin peptides are toxic for inducing mucosal damage or immunogenic to specifically stimulate T cell clones from the patients [29]. Thus, for further clinical application, the medical and toxic effect should also be inspected, and the bioavailability and absorption of it should be evaluated in our future research.

## 4. Materials and Methods

### 4.1. Plant Material and Chemicals

The *Coix* seeds were obtained from Tongrentang (Beijing, China). Pepsin, ACE, hippuryl-l-histidyl-l-leucine (HHL) and hippuric acid (HA) were purchased from Sigma-Aldrich (St. Louis, MO, USA). Acetonitrile, HPLC grade, was supplied by Fisher Scientific (Pittsburgh, PA, USA). Trifluoroacetic acid (TFA, MS grade) was purchased from Merck KGaA (Darmstadt, Germany). All other chemicals and reagents were analytical grade.

### 4.2. Preparation of Enzymatic Hydrolysate of Coix Glutelin

The preparation of CGH was carried out by the method of Yuan [10]. Four fractions of storage protein (albumin, globulin, prolamin and glutelin) were sequentially extracted from the dried *Coix* powder with deionized water, 0.5 M NaCl, 70% ethanol (containing 5% β-mercaptoethanol) and 12.5 mM sodium borate buffer (containing 1% SDS and 2% β-mercaptoethanol), respectively. Glutelin (2%, *w*/*v*) was dissolved in 0.01 M HCl and pepsin was added with enzyme/substrate ratio of 1/10. The mixture was incubated at the temperature of 37 °C for 48 h and then heated (100 °C, 5 min) to end the process of hydrolysis by inactivating the enzyme. The hydrolysate was centrifuged at 10,000 rpm for 10 min and the supernatant was used for further analysis.

### 4.3. Ultrafiltration (UF) of the CGH

The pepsin hydrolysate was firstly filtered through a G4 funnel (80 mm) and then the UF (JM1812-1, Dalian Yidong Membrance engineering equipment Co., Ltd., Dalian, China) was carried out with a 3 kDa molecular weight cut-off system to collect the ultrafiltrate. Four different pressures (0.1, 0.2, 0.3 and 0.4 MPa, respectively) were applied to compare its effect on the ACE inhibitory activity. The permeate fraction was frozen at −80 °C for 24 h and then lyophilized.

### 4.4. Gel Filtration Chromatography (GFC) Separation

The ultrafiltrate was further purified by GFC. After being filtered through a 0.45 μM nylon syringe filter, the sample was applied to a Sephadex G-10 (GE Healthcare, 17-0010-02, Uppsala, Sweden) column (1.6 cm × 80 cm), which was equilibrated with distilled water. Elution was performed with the distilled water at the flow rate of 1 mL·min^−1^. The absorbance of elutes were monitored at 220 nm. The collected fractions were freeze-dried for the determination of ACE inhibitory activity and further purification.

### 4.5. Reverse-Phase High Performance Liquid Chromatography (RP-HPLC) Purification

The active fractions separated by the above method were further purified by a Symmetry PrepTM C_18_ column (7 μm, 7.8 mm × 300 mm, Waters corporation, Milford, MA, USA) equipped with an Agilent series 1100 HPLC instrument (Agilent, California, CA, USA,) with flow rate of 2.0 mL·min^−1^. Peptides were eluted and monitored at 220 nm using the mobile phase composed of solution A (water containing 0.1‰, TFA, *v*/*v*) and solution B (acetonitrile) with a linear gradient of B from 5% to 15% for 30 min, and then the gradient was from 15% to 30% B within 25 min. Major peaks were collected, lyophilized and tested for ACE-inhibitory activities.

### 4.6. Estimation of Peptide Content

The peptide content of each collected fraction was estimated using the Pierce Bicinchoninic Acid (BCA) Protein Assay Kit (Thermo Scientific, San Diego, CA, USA) according to the protocol provided by the manufacturer.

### 4.7. ACE Inhibitory Activity Assay

The ACE inhibitory activity assay using HHL as the substrate and HA as the product was performed by the method described by Yuan et al. [10]:

Inhibitory activity (%) = (A_control_ − A_sample_)/A_control_ × 100%, (1)

where A_control_ is the HA peak area of negative control; and A_sample_ is the HA peak area of the sample. The concentration of peptide fraction that inhibited ACE activity by 50% (IC_50_) was calculated using a non-linear regression from a plot of ACE inhibition versus sample concentrations.

### 4.8. Mass Spectrometry Analysis of ACE-Inhibitory Peptides

To analyze the amino acid sequence of the peptides in the most active fraction, the sample was analyzed by ESI-MS/MS based on a Triple TOF 5600 Mass spectrometer (AB SCIEX, Darmstadt, Germany) fitted with a nanospray III source (AB SCIX) and a pulled quartz tip as the emitter (New, Objectives, Woburn, MA, USA). The parameters were as follows: ion spray voltage, 2.5 kV; curtain gas, 30 psi; nebulizer gas, 15 psi; interface heater temperature, 150 °C. The MS was operated with an RP of greater than or equal to 30,000 full width half maximum (FWHM) for TOF MS scans. For information dependent acquisition (IDA), survey scans were acquired in 100 ms and as many as 40 product ion scans were collected if exceeding a threshold of 150 counts per second (counts/s) and with a 2+ to 5+ charge-state. Dynamic exclusion was set for 1/2 of peak width (15 s), and then the precursor was refreshed off of the exclusion list. Peptide sequences obtained from the MS/MS spectra were confirmed by search for the National Center for Biotechnology Information (NCBI) nonredundant and Uniprot database.

### 4.9. Pharmacophore Mapping of Identified Peptides

The ACE pharmacophore model was firstly employed for predicting the activity of identified peptides. The 3D quantitative pharmacophore hypotheses of ACE was constructed by Zhang [30], which consisted of four features, one hydrogen bond acceptor, one hydrogen bond donor, one negative ionizable, and one hydrophobic aromatic. Three-dimensional conformations of identified peptides were constructed and minimized by Discovery Studio 4.0 (DS, Accelrys Inc., San Diego, CA, USA) within the relative energy threshold of 20.0 kcal·mol^−1^ by the CHARMm force field and BEST mode. The peptide completely mapped with the ACE pharmacophore model was further screened by molecular simulation of ACE-peptide interaction.

### 4.10. Structural Optimization for Peptides by Molecular Docking

Molecular docking was then applied for further analysis of the mapped peptide. The crystal structure of human ACE (PDB: 1O86) [31] and its initial ligand, lisinopril, was used to carry out molecular docking study. This enzyme was automatically cleaned up, including deleting waters, adding hydrogen atoms and building loop regions. The active site of 1O86 was defined by initial ligand lisinopril and the binding pocket size was created with a sphere radius of 10.13 Å around lisinopril present in 1O86.

CDOCKER as well as its corresponding scoring functions were used to implement the molecular docking study. After being extracted from the active pocket, lisinopril was re-docked into 1O86, and the root-mean-square deviation (RMSD) value was 0.98, which indicated that the selected molecular docking module would reproduce the experimentally observed binding mode for ACE inhibitors. The docking score of lisinopril was set to be a reference in analyzing the peptides by molecular docking. Meanwhile, with the default parameters, the peptides which were hit by the ACE pharmacophore were used to perform the docking study.

In order to improve the activity of peptide, in silico proteolysis was further implemented by a dedicated tool in the BIOPEP database (http://www.uwm.edu.pl/biochemia/index.php/pl/biopep). The peptide was obtained by enzymes that are found in the gastrointestinal digestion tract using in silico analysis including pepsin (pH > 1.3), trypsin and chymotrypsin [32]. The hydrolyzed peptides were docked into the active site of 1O86. The key residues between hydrolyzed peptides and ACE of docking poses were also analyzed for identifying the binding mode of peptides.

### 4.11. Synthesis of the Optimized Peptide

The peptide was chemically synthesized by the Beijing Scilight Biotechnology Ltd. Co. (Beijing, China). The percentage purity of the synthesized peptides was greater than 98% as determined by HPLC analysis.

### 4.12. Antihypertensive Effect of Synthesized Peptide in Spontaneously Hypertensive Rats

Male SHRs (9 weeks old, specific pathogen free, SPF) were purchased from Vital River Laboratory Animal Technology Co., Ltd. (Beijing, China). Rats were kept under a 12 h light–dark cycle at 22 ± 4 °C with relative humidity of 50% ± 20%. The standard chow diet and distilled water were provided ad libitum during the experiments. The animals adapted to the new situation for one week before the experiment. Experimental procedures were conducted in strict accordance with the PR China legislation on the use and care of laboratory animals.

SHRs were randomly divided into 5 groups with 6 rats in each group. The heptapeptide GAAGGAF was dissolved in distilled water and SHRs were orally administrated at a dose of 7.5, 15.0 and 30.0 mg/kg BW, respectively, with distilled water and captopril as the control group served as the negative and positive control separately. All of the groups were orally administrated with the volume of 5 mL/kg body weight. The SBP of the rats were measured using the tail-cuff method [17].

### 4.13. Statistical Analysis

All results in this paper were reported as mean ± standard deviation. Significance of differences was determined by the Student’s *t*-test. The *p*-value less than 0.05 was considered to be significant.

## 5. Conclusions

Our present work clearly indicated that the CGH possesses the potential as of anti-hypertension. In this study, six peptides were identified from the sub-fraction F5-3, which was obtained by UF, GFC and RP-HPLC subsequently. Through further molecular simulation screening and a series of division and optimization, the most potent peptide GAAGGAF (IC_50_ = 14.19 μmol·L^−1^) was obtained, and this heptapeptide manifested a satisfactory antihypertensive effect in vivo in SHR. In conclusion, this research proved that GAAGGAF from CGH could be a potential candidate for the development of nutraceuticals and pharmaceuticals active against hypertension and its related diseases.

## Figures and Tables

**Table 1:** Peptides from sub-fraction F5-3 identified by electrospray ionisation tandem mass spectrometry (ESI-MS/MS).

No.	Peptide Sequence	Mr(exp)	Mr(calc)
1	QEKQKL	756.41	756.40
2	EKHNRL	796.44	796.42
3	QSGDQQEF	938.32	938.36
4	VGQLGGAAGGAF	1004.47	1004.49
5	QQQQQQQQQQQQSL	1740.73	1740.76
6	PATAHKQQQQADANMAKL	1966.94	1966.95

**Table 2:** The predicted results of peptides by pharmacophore and docking.

NO. ^a^	Peptide Sequence	Fitvalue ^b^	-CDOCKER ENERGY ^c^	NO. ^a^	Peptide Sequence	Fitvalue ^b^	-CDOCKER ENERGY ^c^
1-1	QEKQKL	none	none	2-4	GGAAGGAF	0.95	165.39
1-2	EKHNRL	none	none	3-1	GAAGGAF	0.96	163.62
1-3	QSGDQQEF	0.97	none	3-2	AAGGAF	0.95	143.18
1-4	VGQLGGAAGGAF	0.96	180.40	3-3	AGGAF	0.96	132.88
1-5	QQQQQQQQQQQQSL	none	none	3-4	GGAF	0.96	119.30
1-6	PATAHKQQQQADANMAKL	none	none	3-5	GAF	0.95	112.38
2-1	VGQL	none	108.65	3-6	AF	0.23	99.54
2-2	GGAAGGA	none	137.75	4	lisinopril	0.95	71.22
2-3	VGQ	none	112.56				

^a^ No. 1-1 to 1-6 represented the sequence of six identified peptides; No. 2-1 to 2-4 represented the sequence of four peptides by in silico proteolysis of VGQLGGAAGGAF; No. 3-1 to 3-6 stood for the sequence of six peptides by sequential division from GGAAGGAF; ^b^ Fitvalues were the scores of pharmacophore screening; ^c^ -CDOCKER ENERGY was the scoring function of docking modelling and represented the interaction ability between ligands and receptor.

## Supplementary Materials

Supplementary materials can be accessed at: http://www.mdpi.com/1420-3049/22/1/123/s1.
