# Novel Antihypertensive Peptides Derived from Adlay (*Coix larchryma-jobi* L. var. *ma-yuen Stapf*) Glutelin

**DOI:** 10.3390/molecules22040534

**Published:** 2017-03-27

**Authors:** Bin Li, Liansheng Qiao, Lingling Li, Yanling Zhang, Kai Li, Lingzhi Wang, Yanjiang Qiao

**Affiliations:** Beijing University of Chinese Medicine, 6, South Zhonghuan Road, Wangjing, Chaoyang District, Beijing 100102, China; libinzyy@163.com (B.L.); b20100222012@163.com (L.Q.); lilingling@126.com (L.L.); collean_zhang@163.com (Y.Z.); lk20162016@163.com (K.L.)

The authors wish to make the following corrections to this paper:

1. Change in Title
  The original title: “A Novel Antihypertensive Derived from Adlay (*Coix larchryma-jobi* L. var. *ma-yuen Stapf*) Glutelin” should be corrected as: “Novel Antihypertensive Peptides Derived from Adlay (*Coix larchryma-jobi* L. var. *ma-yuen Stapf*) Glutelin”.
2. Change in Main Body Paragraphs
  There are three errors in this article [1]:
  (1) Section 2.6. The ACE Inhibitory Activity of GAAGGAF In Vitro should be corrected to 2.6. The Antihypertensive Effect of GAAGGAF In Vivo.
  (2) Section 4.10 and 4.11 swap places
  (3) The caption Figure 3. Representation of the angiotensin converting enzyme (ACE) pharmacophore model. Purple sphere indicated hydrogen bond donor, green sphere indicated hydrogen bond acceptor, light blue sphere indicated hydrophobic aromatic features, and dark blue sphere indicated negative ionizable. (**A**) represented the best pharmacophore model of ACE; (**B**) represented the ACE pharmacophore model mapped with GAAGGAF. Molecular interactions between PDB 1O86 and the ligands: (**C**) Lisinopril; and (**D**) GAAGGAF should be corrected to Figure 3. Representation of the angiotensin converting enzyme (ACE) pharmacophore model and molecular interactions between PDB 1O86 and the ligands. Purple spheres indicate hydrogen bond donors, green spheres indicate hydrogen bond acceptors, light blue spheres indicate hydrophobic aromatic features, and dark blue spheres indicate negative ionizable. (**A**) The best pharmacophore model of ACE; (**B**) the ACE pharmacophore model mapped with GAAGGAF; (**C**) molecular interaction between 1O86 and lisinopril; (**D**) molecular interaction between 1O86 and GAAGGAF.

The authors would like to apologize for any inconvenience caused to the readers by these changes.
