# Supplementary material for: Novel Antihypertensive Peptides Derived from Adlay (Coix larchryma-jobi L. var. ma-yuen Stapf) Glutelin
Source: Molecules. 2017 Jan 13;22(1):123. doi: 10.3390/molecules22010123 (PMC6155628; doi:10.3390/molecules22010123)
Supplement: Supplementary file 1 [file molecules-22-00123-s001.pdf]

Supplementary Materials: A Novel Antihypertensive Derived from Adlay (*Coix larchryma-jobi* L. var. *ma-yuen Stapf*) Glutelin

Bin Li, Liansheng Qiao, Lingling Li, Yanling Zhang, Kai Li, Lingzhi Wang and Yanjiang Qiao

The peaks of HA and HHL standard eluted out of C_{18} column were showed in Figure S1A,B, respectively. The negative control peak of borate buffer was showed in Figure S1 and the positive control of Captopril demonstrated a significant ACE inhibitory ratio of 86.43% at a concentration of 2 × 10^{-8} mol·L^{-1} (Figure 1D). The HA concentration of the CGH (Figure 1E) sharply decreased compared with the negative control.

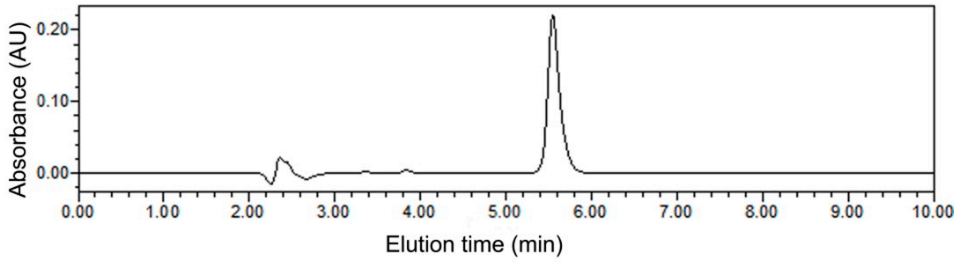

(**A**)

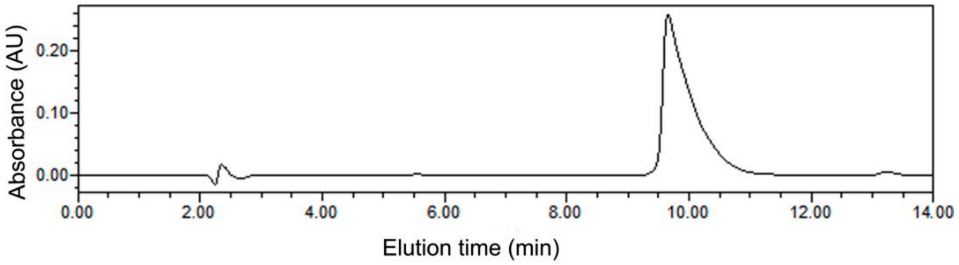

(**B**)

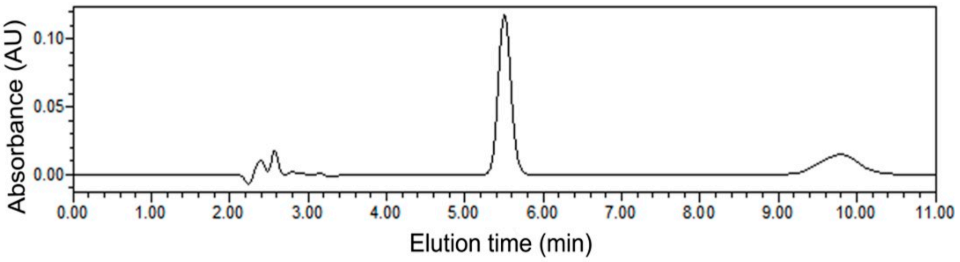

(**C**)

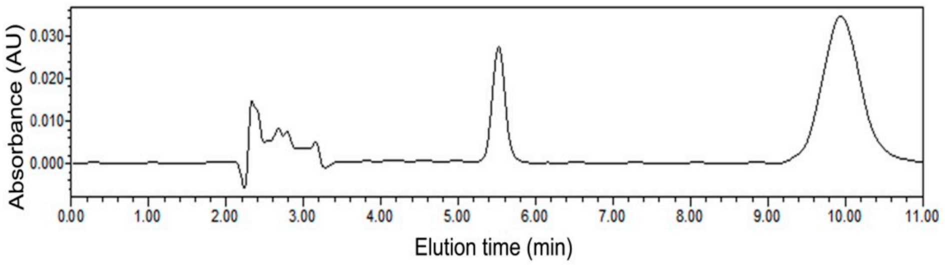

(**D**)

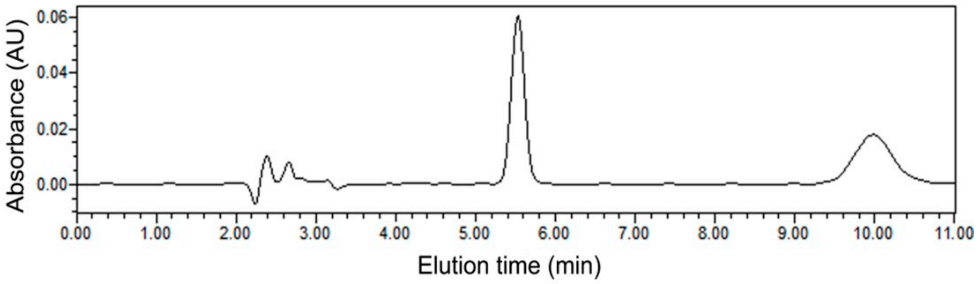

(**E**)

Figure S1. RP-HPLC chromatograms of (**A**) HA standard; (**B**) HHL standard; (**C**) blank control; (**D**) captopril of 2 × 10^{-8} mol·L^{-1}; and (**E**) the CGH (≤3 kDa) obtained under 0.1 Mpa UF pressure (final concentration of 0.02 mg·L^{-1}).

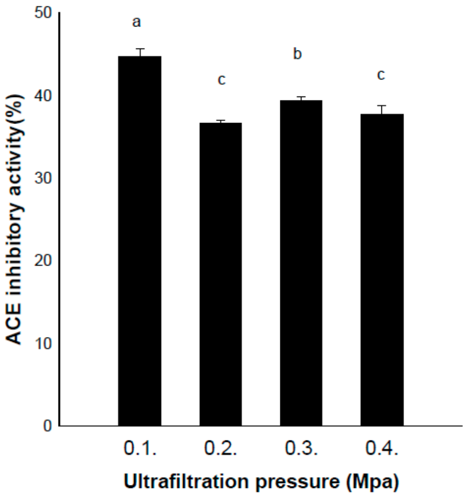

Figure S2. The ACE inhibitory activity of CGH (≤3 KDa) obtained under different pressure. The samples were measured at the final concentration of 0.02 mg·L^{-1}. Different letters above the column indicated the percentage of ACE-inhibitory activity were significantly different ($p < 0.05$).

The ACE Inhibitory Activity of Fractions F1–F5

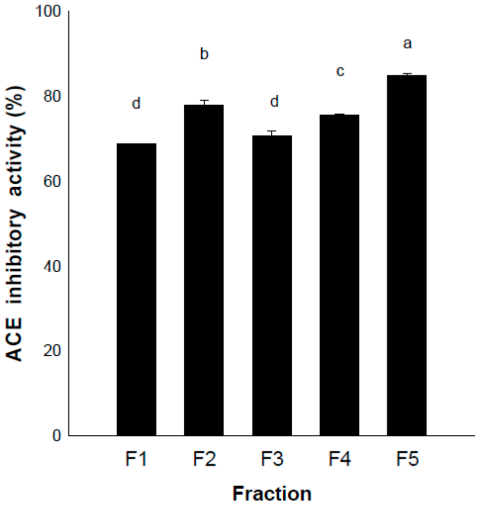

Figure S3. The ACE inhibitory activity of fractions separated by GFC. Different letters above the column indicated the percentage of ACE-inhibitory activity were significantly different ($p < 0.05$).

Sequence Profile of the Peptides VGQLGGAAGGAF

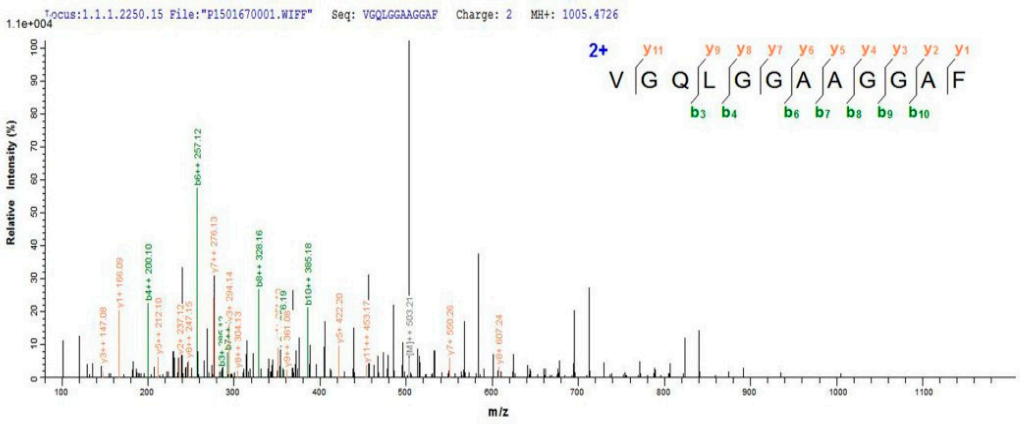

Figure S4. The ESI-MS/MS spectrum of VGQLGGAAGGAF.

The ACE Inhibitory Activity of Fractions F5-1–F5-9

Table S1. ACE inhibition rate of sub-fractions separated by RP-HPLC.

Fractions	ACE Inhibitory Rate (%)
F5-1	42.14 ± 0.48 [c]
F5-2	50.42 ± 1.81 [b]
F5-3	60.06 ± 0.72 [a]
F5-4	3.39 ± 2.07 [g]
F5-5	31.22 ± 0.71 [d]
F5-6	30.18 ± 2.01 [d]
F5-7	14.91 ± 2.73 [e]
F5-8	41.10 ± 1.26 [c]
F5-9	9.61 ± 3.21 [f]

The final sample concentration was 0.01 mg·mL^{-1}. The data were expressed as mean ± standard deviation (n = 3). Different letters (the superscript words a–g) indicated above the percentage of ACE inhibitory activity were significantly different ($p < 0.05$).
